# An innovative gene expression modulating strategy by converting nucleic acids into HNC therapeutics using carrier-free nanoparticles

**DOI:** 10.3389/fimmu.2023.1343428

**Published:** 2024-01-11

**Authors:** Heyuan Liu, Yinong Huang, Zongfang Li, Suxia Han, Tianya Liu, Qian Zhao

**Affiliations:** ^1^ National and Local Joint Engineering Research Center of Biodiagnosis and Biotherapy, The Second Affiliated Hospital of Xi’an Jiaotong University, Xi’an, China; ^2^ Shaanxi Institute of Pediatric Diseases, Xi’an Children’s Hospital, Xi’an, China; ^3^ Department of Radiation Oncology, The First Affiliated Hospital of Xi’an Jiaotong University, Xi’an, China; ^4^ Institute for Stem Cell and Regenerative Medicine, The Second Affiliated Hospital of Xi’an Jiaotong University, Xi’an, China; ^5^ Department of Otorhinolaryngology Head and Neck Surgery, The First Affiliated Hospital of Xi’an Jiaotong University, Xi’an, China

**Keywords:** derivatives of siRNA, automatic assembly system, siRNA clinical translation, carrier-free nanoparticles, anticancer therapeutics

## Abstract

**Background:**

Cell fate and microenvironmental changes resulting from aberrant expression of specific proteins in tumors are one of the major causes of inadequate anti-tumor immune response and poor prognosis in head and neck cancer (HNC). Eukaryotic initiation factor 3C (eIF3c) has emerged as a promising therapeutic target for HNC due to its ability to regulate protein expression levels in tumor cells, but its drug development is difficult to achieve by targeting traditional protein-protein interactions. siRNA has emerged as a highly promising modality for drug development targeting eIF3c, while its application is hindered by challenges pertaining to inadequate stability and insufficient concentration specifically within tumor sites.

**Method:**

We employed a method to convert flexible siRNAs into stable and biologically active infinite Auric-sulfhydryl coordination supramolecular siRNAs (IacsRNAs). Through coordinated self-assembly, we successfully transformed eIF3C siRNAs into the carrier-free HNC nanotherapeutic agent Iacs-eif3c-RNA. The efficacy of this agent was evaluated *in vivo* using HNC xenograft models, demonstrating promising antitumor effects.

**Results:**

Iacs-eif3c-RNA demonstrated the ability to overcome the pharmacological obstacle associated with targeting eIF3C, resulting in a significant reduction in eIF3C expression within tumor tissues, as well as effective tumor cell proliferating suppression and apoptosis promotion. In comparison to monotherapy utilizing the chemotherapeutic agent cisplatin, Iacs-eif3c-RNA exhibited superior anti-tumor efficacy and favorable biosafety.

**Conclusion:**

The utilization of Iacs-eif3c-RNA as a carrier-free nanotherapeutic agent presents a promising and innovative approach for addressing HNC treating challenges. Moreover, this strategy demonstrates potential for the translation of therapeutic siRNAs into clinical drugs, extending its applicability to the treatment of other cancers and various diseases.

## 1 Introduction

Head and neck carcinoma (HNC) is a prevalent category of cancers, primarily consisting of squamous cell carcinomas, which account for over 90% of malignant cases (1, 2). A significant majority of patients, approximately 70%, are diagnosed with intermediate to advanced stages, resulting in an unsatisfactory 5-year survival rate of less than half (1, 3, 4). Regrettably, the prognosis for HNC has not shown notable improvement in recent years, largely attributed to the constraints imposed by the available treatment modalities (5). The issue of resistance to primary treatments for HNC, including chemotherapy (e.g. cisplatin) and immunotherapy, constitutes a significant determinant of prognosis (6, 7). In light of the groundbreaking therapeutic advancements facilitated by immune checkpoint antibodies and other therapies reliant on protein-protein interactions (PPIs), it is noteworthy that only a minority of patients with HNC exhibit positive responses (8–10). This phenomenon can potentially be attributed to the abnormal expression of certain proteins in tumor cells, which consequently impairs immune cell functionality, leading to tumor immune evasion and immunosuppression (11–14). Noteworthily, the investigation of eukaryotic initiation factor 3c (eIF3C) as a potential antitumor target in the therapeutic exploration of HNC has been progressively undertaken (15, 16). eIF3C, a constituent of the largest eukaryotic translation initiation factor eIF3 complex, plays a crucial role in regulating transcript-specific translation during development and exhibits elevated expression levels in various cancer types (15, 17, 18). Suppression of eIF3C results in disruption of translation initiation complexes and decreased protein expressions, leading to cell cycle arrest and apoptosis (15, 16, 19). Nevertheless, similar to other tumor-specific proteins and associated PPIs, inhibiting eIF3C through direct protein-level regulation poses significant challenges.

In the past few years, there has been a rise in cancer therapeutic approaches that focus on addressing aberrant target protein levels in tumor cells (20–22). Among these approaches, small interfering RNAs (siRNAs) have shown potential in rectifying the expression of specific genes, thereby offering a promising avenue for tumor therapy (23–26). Unlike peptides and protein-based drugs, which possess intricate interfaces connecting multiple protein structural features, siRNAs directly suppress targets on the pre-expression level, thereby impeding the development of tumor-promoting PPIs (27, 28). Moreover, the therapeutic strategies involving siRNA exhibit the capability to reach intracellular targets, a feat that proves challenging for drugs based on PPIs (29). Therefore, it is promising to show beneficial effects in anti-tumor immunity by reducing the levels of proteins involved in immune escape through siRNAs. Nevertheless, siRNAs suffer from inadequate stabilization, susceptibility to degradation, absence of tumor targeting, and confront obstacles in terms of applicability (25, 26). Naked siRNAs, in particular, are subject to ineffective depletion due to endonuclease degradation, nonspecific uptake by macrophages, charge repulsion, and other physiological barriers, resulting in insufficient concentration at the tumor site (26, 30, 31). Consequently, there is a pressing necessity to devise a strategy for transforming therapeutic siRNAs into viable clinical drugs to address the treatment of HNC.

**Figure 1:**
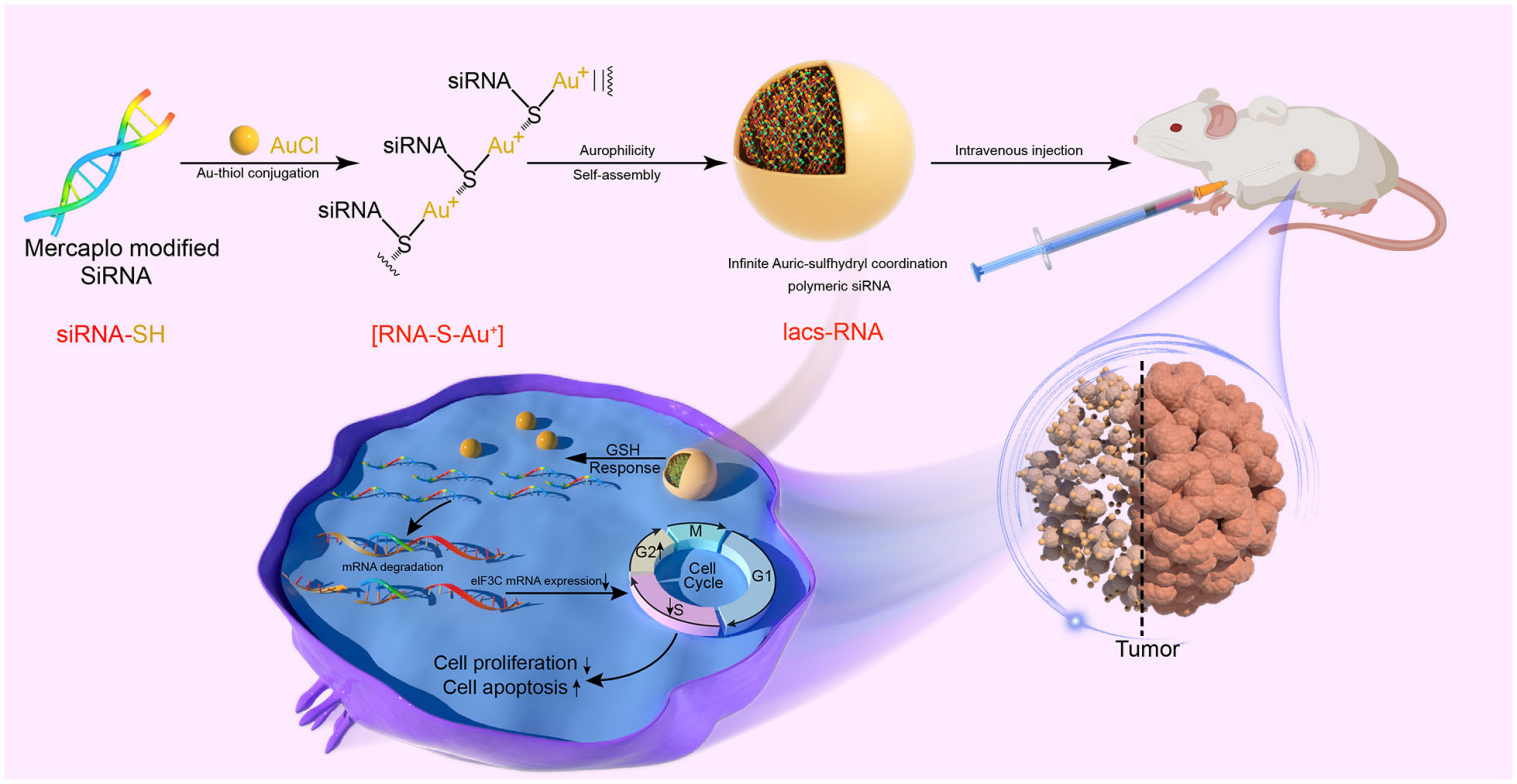
Fabrication and therapeutic mechanism of Iacs-eif3c-RNA.

Herein, we present a comprehensive approach to transform flexible siRNAs into stable and biologically active infinite Auric-sulfhydryl coordination supramolecular siRNA (IacsRNA), using a mild and straightforward chemical pathway, building upon prior research (32, 33). By employing this methodology, we successfully converted eIF3C siRNAs into an HNC therapeutic agent, exhibiting anti-tumor efficacy both in cellular and animal models. Specifically, Iacs-eif3c-RNA precursors, prepared by the reaction of mercapto-modified eIF3C siRNA with gold, was binding with each other to assemble into carrier-free nano-particles driven by gold-thioether coordination, called Iacs-eif3c-RNA (**Figure 1**). Through a xenograft murine model of head and neck squamous cell carcinoma (HNSCC), it is successfully demonstrated that Iacs-eif3c-RNA effectively overcame the pharmacological barrier associated with eIF3C, significantly reduced the expression level of eIF3C in tumor tissues and exhibited inhibitory effects on tumor growth *in vivo*. Furthermore, it is revealed that in HNSCC xenograft mouse models constructed with cisplatin-resistant cell line, Iacs-eif3c-RNA demonstrated superior anti-tumor efficacy and remarkable biosafety compared to cisplatin. This work provides a novel and effective carrier-free nucleic acid nanotherapeutic agent for the treatment of HNC, and proposes a promising approach for the translation of therapeutic siRNAs into potential clinical drugs for the management of cancer and other ailments.

## 2 Results

### 2.1 Evaluation of siRNA eIF3C silencing target gene

**Figure 2:**
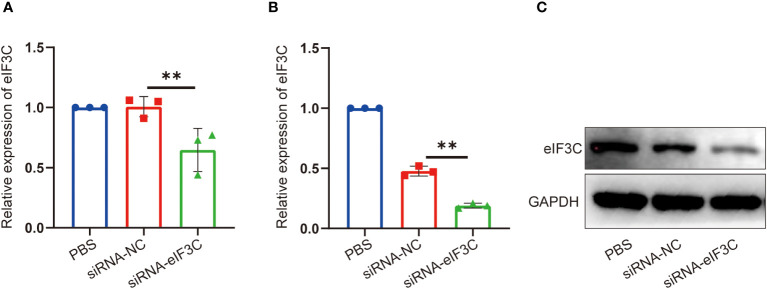
The expression of eIF3C in 5-8F and FaDu cells decreased after siRNA-eIF3C treatment. **(A)** The expression of eIF3C mRNA in 5-8F cells. **(B)** The expression of eIF3C mRNA in FaDu cells. **(C)** The expression of eIF3C protein in FaDu cells. The data were presented as mean ± s.d. **, *p*<0.01.

In order to clear the eIF3C siRNA interference effect on eIF3C expression in head and neck carcinoma, eIF3C siRNA (siRNA-eIF3C) and the negative control siRNA (siRNA-NC) encapsulated in liposome were used to infect the human FaDu cell line and 5-8F cell line, both of which are from HNC. Compared with those of negative control, the gene expression of eIF3C was decreased remarkably in both FaDu and 5-8F cells (**Figures 2A, B**), and the protein expression of eIF3C was inhibited significantly in FaDu cells (**Figure 2C**) after eIF3C siRNA treatment. These results indicated that ability of eIF3C siRNA to interfere with target gene expression implying eIF3C siRNA could be used to prepare Iacs-eif3c-RNA by reacting with HAuCl_4_.

### 2.2 Design and synthesis of Iacs-eif3c-RNA

**Figure 3:**
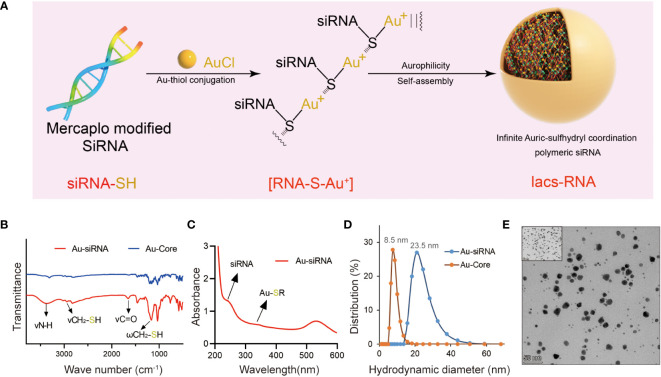
Fabrication and characteristic of Iacs-eif3c-RNA. **(A)** The preparation process of Iacs-eif3c-RNA. **(B)** FT-IR spectra of Iacs-eif3c-RNA and Au-Core. The band at 1200 cm^−1^ which was attributed to the stretching vibration of -SH. **(C)** UV-Vis absorption spectra of Iacs-eif3c-RNA. The distinct absorption peaks at 330 nm in the UV-Vis region is the absorption peaks for the Au-S-siRNA species. **(D)** Hydrodynamic diameter distributions of Iacs-eif3c-RNA and Au-Core. **(E)** Transmission electron micrograph images (TEM) of Iacs-eif3c-RNA (scale bar = 50 nm).

In the combination reaction, ionized HAuCl_4_ (Au^3+^) banded with the thiol group of thiol-siRNA eIF3C (eIF3C siRNA-SH) to produce Iacs-eif3c-RNA precursor, then the precursor connected to each other through auric-sulfhydryl coordination to form Iacs-eif3c-RNA, eventually (**Figure 3A**).

In order to determine whether polymer precursors were formed, FT-IR and UV-vis spectroscopy of above reactions were implemented. A prominent increased absorption peak appeared at 1200 cm^-1^ in FT-IR, belong to fingerprint, indicated the change of molecular structure implying the form of Iacs-eif3c-RNA precursor (**Figure 3B**). In UV-vis, an absorption peak at 330 nm was found in the compound of HAuCl_4_ and mercapto modified siRNA, demonstrated that Iacs-eif3c-RNA precursor was obtained successfully for the characteristic peak of Au^+^-SR absorption appearance (**Figure 3C**). In order to sure the Iacs-eif3c-RNA made of above precursor was taken shape, dynamic light scattering and transmission electron microscope (TEM) were implemented. The hydrodynamic diameter of Iacs-eif3c-RNA peaked at ~23.5 nm measured by dynamic light scattering, implying formation of nanoscale clusters (**Figure 3D**). Moreover, in TEM images, Iacs-eif3c-RNA presented similar particle characteristics further supporting dynamic light scattering results (**Figure 3E**). Through the above method and detection, we successfully converted eIF3C siRNA into stable Iacs-eif3c-RNA.

### 2.3 The biosafety properties of Iacs-eif3c-RNA *in vivo*

**Figure 4:**
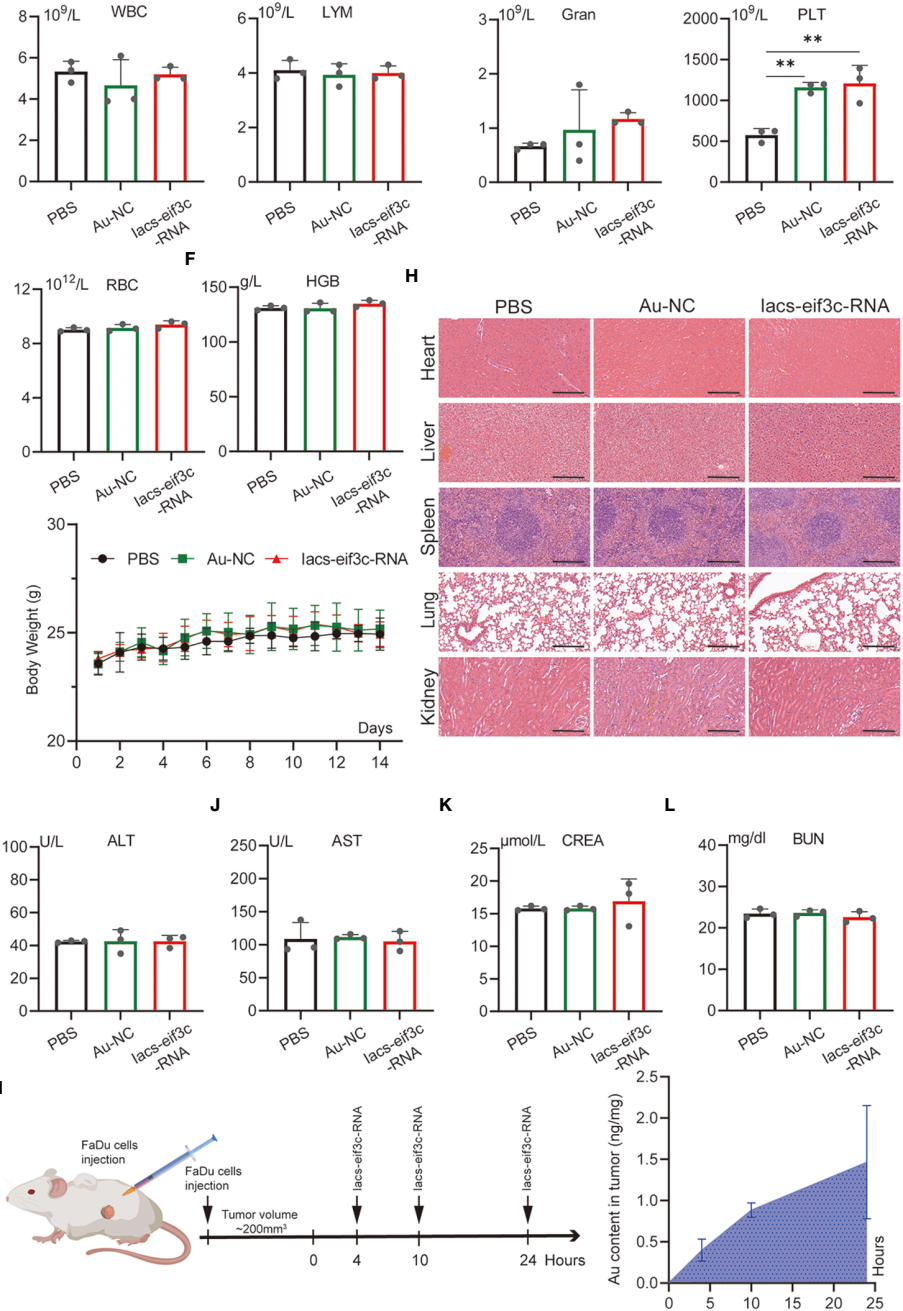
*In vivo* safety evaluation of Iacs-eif3c-RNA. **(A–F)** White blood cell (WBC), lymph (LYM), granulocyte (Gran), hemoglobin (HGB), red blood cell (RBC), and platelet (PLT) in peripheral blood reflecting blood metabolic capacity. **(G)** The body weight of mice during 14-day treatments. **(H)** The representative histological H&E staining images of brain, heart, liver, lung, spleen, kidney in mice after the indicated treatments (magnification: 20×, scale bar: 200µm). **(I–L)** Alanine aminotransferase (ALT), aspartate aminotransferase (AST), creatinine (CREA) and urea (UREA) were measured to reflect liver function. **(M)** The content of Au ion in tumor detected by Inductively Coupled Plasma Mass Spectrometer. Tumor tissue of mice was collected at 0, 4, 10 and 24 hours after Iacs-eif3c-RNA injected intraperitoneally. The data were presented as mean ± s.d. **, *p*<0.01.

To evaluate the toxicity of the Iacs-eif3c-RNA *in vivo*, an extensive study of toxicity was conducted using C57BL/6 mice. Iacs-eif3c-RNA and negative control (Au-NC) were intraperitoneally injected every other day, undergoing a 14-day administration. On the 15^th^ day, mice were sacrificed for the following experiment. The results of blood routine tests presented that the granulocyte (Gran) and platelet (PLT) increased within normal limits (Gran: 0.23-3.6×10^9^/L; PLT: 400-1600×10^9^/L) in peripheral blood after Iacs-eif3c-RNA treatment (**Figures 4C, D**), while the white blood cell (WBC), lymph (LYM), hemoglobin (HGB) and red blood cell (RBC) didn’t appear significant differences (**Figures 4A, B, E, F**). It is identified that the effects of Iacs-eif3c-RNA on hematologic systems have been controlled within safe limits. Body weight of mice among three groups exhibited consistent growth trends during the administration (**Figure 4G**), implying that Iacs-eif3c-RNA did not adversely affect normal physiological activities such as feeding in mice. In order to confirmed whether Iacs-eif3c-RNA affected organs, the indicators of organ damage were measured. The histological H&E staining of heart, liver, kidney, lung and spleen also showed normal cell morphology (**Figure 4H**). Alanine aminotransferase (ALT), aspartate aminotransferase (AST), creatinine (CREA) and blood urea nitrogen (BUN) related to liver and kidney function also have similar level among three groups (**Figures 4I–L**). The above results indicated that Iacs-eif3c-RNA possessed favorable biosafety and the potential for clinical application.

### 2.4 Iacs-eif3c-RNA achieved tumor eIF3C expression disruption and efficient tumor growth suppression *in vivo*

**Figure 5:**
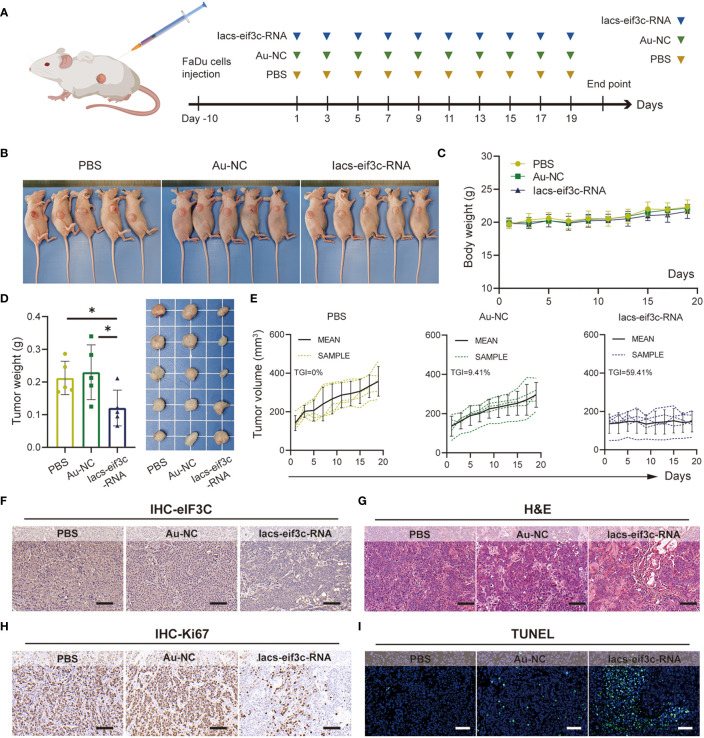
Iacs-eif3c-RNA inhibited the tumor growth *in vivo*. **(A)** Schematic plot of this test. **(B)** The photos of mice with tumor **(C)** The body weight recorded during Iacs-eif3c-RNA injected intraperitoneally every two days. **(D)** The photos and weight of tumor tissues recorded on the 20^th^ day after Iacs-eif3c-RNA injected intraperitoneally. **(E)** The tumor volume of mice recorded during Iacs-eif3c-RNA injected intraperitoneally every two days. **(F-I)** The representative histological H&E staining, TUNEL staining and IHC staining (eIF3C and ki67) of tumor executed after mice treated with Iacs-eif3c-RNA (magnification: 20×, scale bar: 100µm). The data were presented as mean ± s.d. *, *p*<0.05.

To determine whether nanoparticles are able to target to and accumulate in tumor site, we measured the content of gold in tumor tissue of mice, which were injected with Iacs-eif3c-RNA after 0, 4, 10 and 24 hours. The result showed that gold content in tumor increased with time (**Figure 4M**), which indicated the Iacs-eif3c-RNA could target to and accumulate in tumor at least 24 hours. To verify the antitumor ability of Iacs-eif3c-RNA, the drugs was intraperitoneally injected to HNC xenograft model every other day in a 19-day cycle (**Figure 5A**). Tumor weights were significantly reduced in mice treated with Iacs-eif3c-RNA, supporting by the photographs of tumors and tumor-bearing mice (**Figures 5B, D**). Mice injected with Iacs-eif3c-RNA showed no abnormalities on body weight during the administration (**Figure 5C**). Encouragingly, Iacs-eif3c-RNA treatment substantially suppressed tumor growth (TGI=59.41%), based on the evidences on volume and H&E staining of tumor tissues (**Figures 5E, G**). Immunohistochemical results indicated that the expression level of eIF3C in tumor tissues treated with Iacs-eif3c-RNA was significantly reduced, which proved that Iacs-eif3c-RNA enriched in tumor tissues and fully played the role of silencing target genes (**Figure 5F**). In addition, in the presence of diminished eIF3C levels, expression of Ki67 lowered in Iacs-eif3c-RNA-treated tumor tissues, and the number of TdT-mediated dUTP nick end labeling (TUNEL) positive cells increased, suggesting that this treatment suppressed tumor cell proliferation and augmented apoptosis (**Figures 5H, I**). And it was interesting that Iacs-eif3c-RNA decreased PD-L1 expression of tumor cells, implying Iac-eif3c-RNA may be benefit to inhibition of immune escape in antitumor immunity (**Figure S1**). Collectively, the results indicated Iacs-eif3c-RNA exerted excellent antitumor efficacy by reducing the intracellular level of eIF3C, inhibiting cell growth and promoting apoptosis, suggesting that it has a sustainable tumor suppressor potential *in vivo*.

### 2.5 Reduced intracellular expression levels of eIF3C exhibited sensitization to chemotherapy in the HNC xenograft model

**Figure 6:**
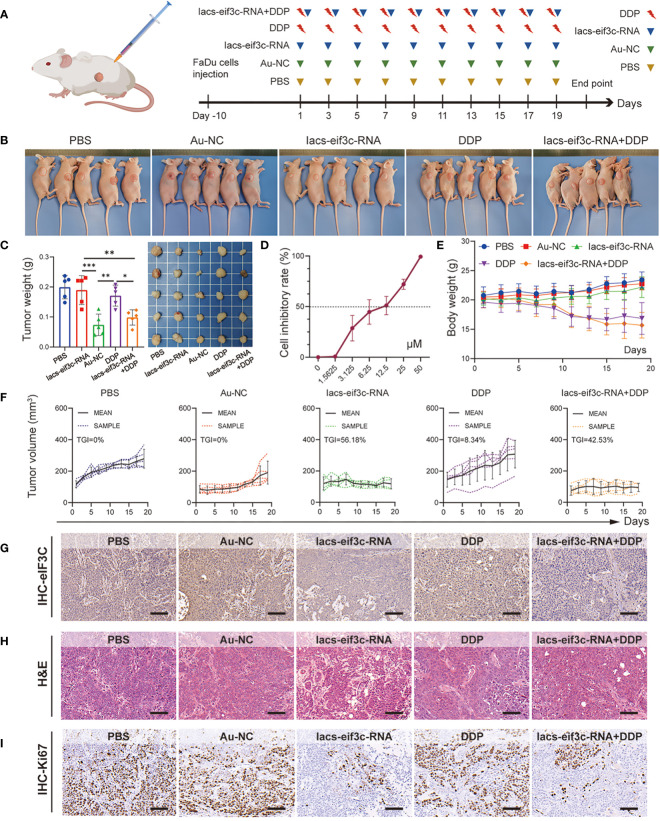
Iacs-eif3c-RNA eIF3C combined with DDP inhibited tumor growth *in vivo*. **(A)** Schematic plot of this test. **(B, C)** The photos of mice and tumor being taken, and the weight of tumor recorded at the 20th day after Iacs-eif3c-RNA injected intraperitoneally. **(D)** The cell inhibitory rate of FaDu cell to DDP. **(E)** The body weight and **(F)** tumor volume of mice recorded during Iacs-eif3c-RNA injected intraperitoneally every two days. **(G–I)** The representative histological H&E staining and IHC staining (eIF3C and ki67) of tumor were executed after mice treated with Iacs-eif3c-RNA (magnification: 20×, scale bar: 100µm). The data were presented as mean ± s.d. *, *p*<0.05; **, *p*<0.01; ***, *p*<0.001.

Chemotherapy is a core measure in the treatment of HNC; in clinical practice, however, chemotherapy tolerance is frequently observed, leading to the unfavorable prognosis for HNC (34, 35). The excellent anti-tumor performance of Iacs-eif3c-RNA, as well as the important molecular biological function of eIF3C itself compelled us to explore its role in the treatment of chemotherapy-resistant HNC. Here, we constructed a HNC xenograft model using cisplatin (DDP)-insensitive FaDu cells to examine the anti-tumor ability of Iacs-eif3c-RNA. The mice were intraperitoneally injected with PBS, Au-NC, Iacs-eif3c-RNA, DDP or a combination of Iacs-eif3c-RNA and DDP every other day during 19-day administration period (**Figure 6A**). The photographic results showed that tumor was smaller after treated with no matter Iacs-eif3c-RNA alone or a combination of Iacs-eif3c-RNA with DDP (**Figures 6B, C**). Both the Iacs-eif3c-RNA monotherapy and combination therapy showed evident tumor suppression, while DDP alone did not significantly reduce tumor weight (**Figure 6C**). The possible explanation for this phenomenon could be attributed to the resistance towards DDP of the FaDu cell line utilized for constructing the tumor model in this study, as evidenced by the IC50 to DDP range range of 6.25 to 12.5 μM. (**Figure 6D**) (36, 37). Iacs-eif3c-RNA inhibited tumor growth (TGI=56.18%) with little impact on body weights, while the combined treatment showed tumor growth inhibition (TGI=42.53%) accompanying body weight decline (**Figures 6E, F**).

It is worth mentioning that the eIF3C level displayed a decline on the basis of Iacs-eif3c-RNA administration, with or without DDP, suggesting that the modulatory effect of Iacs-eif3c-RNA on eIF3C was not affected by DDP-insensitive properties (**Figure 6G**). Meanwhile, this trend was also consistent with the inhibitory effect of Iacs-eif3c-RNA on tumor growth, which was likewise further supported by the results of H&E staining (**Figure 6H**). Ki67 immunohistochemical staining demonstrated that in the absence of an obvious impact under DDP monotreatment, both Iacs-eif3c-RNA and the combined treatment exhibited an inhibitory effect on tumor cell proliferation (**Figure 6I**). The safety of Iacs-eif3c-RNA was further confirmed by H&E staining of internal organs including brain, heart, liver, lung, spleen and kidney (**Figure S2**). In summary, the results demonstrated that Iacs-eif3c-RNA could achieve cisplatin-insensitive tumor growth inhibition in HNC without causing damage to normal tissues and organs, which implies that it has the potential to be a sensitizer or an alternative therapeutic agent in the case of tolerance to conventional chemotherapeutic agents.

## 3 Discussion

The main reason for the poor prognosis of HNC may be due to its risk for relapse and drug resistance (38, 39). For example, the prevalence of drug resistance to cisplatin, a frequently employed chemotherapy agent in the clinical management, was observed to be common during HNC treatment, with a subset of patients exhibiting unresponsiveness to this drug (4, 40, 41). In the last decade, targeted therapeutic drugs with precise positioning and low toxicity provide a gaining interest and promising solution for the treatment of a variety of tumors, including HNC (42–44). Several targeted drugs, such as cetuximab and pembrolizumab, have showed excellent therapeutic potential in the treatment of HNC at present (45–47). As an important subunit of translation initiation factors, eIF3C is positively correlated with Hedgehog signaling pathway (17, 48), which participated in the immunoevasion of certain tumors (49, 50). eIF3C was also involved in the regulation of a variety of human cancers, such as ovarian cancer (18), lung adenocarcinoma (51), and renal cell carcinoma (52). For all the above reasons, eIF3C have been considered to be a promising therapeutic target for HNC (16). Utilizing eIF3C siRNA as a foundation, we have successfully designed and synthesized the targeted therapeutic agent Iacs-eif3c-RNA. (**Figures 2**, **3**) In contrast to peptide and protein derived drugs that possess intricate structures and mechanisms, Iacs-eif3c-RNA exhibited a remarkable ability to suppress tumor growth in the HNC xenograft model by effectively downregulating the gene expression of eIF3C. (**Figure 5**) The xenograft models constructed from cisplatin-resistant HSNCC cell lines exhibited notable weight reduction subsequent to cisplatin administration, indicating the associated systemic toxicity. Conversely, the utilization of Iacs-eif3c-RNA demonstrated an enhanced therapeutic outcome in terms of inhibiting tumor growth, while not inducing any obvious toxicity symptoms. (**Figure 6**) This finding presents a novel and efficacious approach for HNC management.

As early as 2012, the proposal to develop siRNA-based drugs for HNC treating was put forth, which achieved significant anti-tumor effects (53–56). These siRNA drugs function by targeting specific genes, thereby disrupting the expression of target proteins in tumors, leading to cell apoptosis and the modification of the tumor microenvironment to achieve precise tumor suppression. Nevertheless, the clinical application of siRNA-based therapies necessitates considerations of biological activity, transfection efficiency, and biological safety (26). Naked siRNA, when administered intravenously, lacks protection and is susceptible to degradation by endonucleases (26, 30). Additionally, the non-specific uptake of macrophages within the reticuloendothelial system can lead to phagocytic damage to siRNA (26). Within the interstitium, various factors, including the extracellular matrix, charge repulsion between siRNA and the cell membrane, and physiological barriers such as tight junctions, hinder the penetration of siRNA into tumor tissues (31). In the event that siRNA fails to achieve early endosomal escape upon cellular entry, it undergoes acidification and degradation by lysosomes, ultimately resulting in exocytosis (57). In recent years, there has been a continuous effort to enhance the efficacy of siRNA as clinical drugs by improving and updating the methods for preparing siRNA-enveloped nano systems (25, 32, 33, 58, 59). In this study, we used simple reactants to obtain Iacs-eif3c-RNA through a mild preparation method, which ensured the biological activity and improved the stability of siRNA effectively. (**Figure 3**) Encouragingly, our findings suggested that Iacs-eif3c-RNA successfully suppressed the expression of the target gene eif3c *in vivo*, leading to the tumor growth inhibition (**Figures 5**, **6**). In addition, Iacs-eif3c-RNA demonstrated no detrimental effects in terms of body weight, blood indexes, and organ tissue sections, indicating its favorable biosafety and potential for clinical application. (**Figure 4** and **Figure S2**) In conclusion, the utilization of Iacs-eif3c-RNA offers a reliable approach for therapeutic agent development for HNC. Moreover, our study presents a straightforward, effective, and secure strategy for the clinical application of other siRNA molecules.

## Data availability statement

The raw data supporting the conclusions of this article will be made available by the authors, without undue reservation.

## Ethics statement

The animal study was approved by Medical Ethics Committee of Xi’an Jiaotong University. The study was conducted in accordance with the local legislation and institutional requirements.

## Author contributions

HL: Data curation, Formal Analysis, Investigation, Methodology, Software, Visualization, Writing – original draft. YH: Conceptualization, Formal Analysis, Investigation, Methodology, Supervision, Validation, Visualization, Writing – review & editing. ZL: Supervision, Writing – review & editing. SH: Project administration, Supervision, Writing – review & editing. TL: Conceptualization, Funding acquisition, Methodology, Project administration, Supervision, Writing – review & editing. QZ: Conceptualization, Data curation, Project administration, Resources, Supervision, Validation, Writing – original draft.
