# Supplementary material for: An innovative gene expression modulating strategy by converting nucleic acids into HNC therapeutics using carrier-free nanoparticles
Source: Front Immunol. 2024 Jan 11;14:1343428. doi: 10.3389/fimmu.2023.1343428 (PMC10808498; doi:10.3389/fimmu.2023.1343428)
Supplement: Supplementary file 1 [file DataSheet_1.docx]

Supplementary Material

# 1 Method

## 1.1 General remarks

2 OD mercapto modified eIF3C siRNA and negative control siRNA were purchased from GenePharma (Shanghai, China). HAuCl4·XH2O was purchased from Aladdin Chemicals. HAuCl4·XH2O was purchased from Aladdin Chemicals. In this study, Sigma-Aldrich provided the chemicals used unless otherwise specified.

## 1.2 Cell culture and eIF3C siRNA infection

Human nasopharyngeal carcinoma cell lines, 5-8F and FaDu, were acquired from the Chinese Academy of Science cell bank, and were cultured in DMEM supplemented with 10% FBS and 1% Pen Strep at 37℃ in 5% CO_2_. 1×10^6^ 5-8F and FaDu cells were plated into 6-well plates, and then were infected with 75 pmol eIF3C siRNA aided by Lipofectamine 3000 reagent per well. The eIF3C mRNA expression and protein expression was detected at 48 h and 72 h after siRNA infecting, respectively.

The sequences of upstream and downstream primers used in real time quantitative polymerase chain reaction (RT-qPCR) were as follow: (*eIF3C*) F: 5’- AGATGAGGATGAGGATGAGGAC-3’, R: 5’- GGAATCGGAAGATGTGGAACC-3’; (*GAPDH*) F: 5’- TGACTTCAACAGCGACACCCA-3’, R: 5’- CACCCTGTTGCTGTAGCCAAA-3’. The protein expression of eIF3C and GAPDH was detected by western blot with antibodies against eIF3C (1:1000) and GAPDH (1:5000).

## 1.3 Fabrication and physicochemical properties of Iacs-eif3c-RNA

2 OD mercapto modified eIF3C siRNA and negative control siRNA were dissolved in 10 ml HEPES buffer (50mM, pH 7.4) at 50℃. 500 μL 10 mM HAuCl_4_ was added into the siRNA-containing buffer. And then continue whisking above mixture until the solution changes to deep purple-aubergine from golden yellow, which suggested the successful fabrication of infinite Auric-sulfhydryl coordination polymeric siRNA (IacsRNA).

The microstructure of IacsRNA was observed through transmission electron microscopy (TEM) performed on an HT7700. Fourier transform infrared (FT-IR) spectroscopy (Nicolet 6700) and UV−vis absorption spectra (Shimadzu 3000 spectrophotometer) were used to evaluate the chemical structure of IacsRNA. Hydrodynamic particle size distribution was acquired through dynamic light scattering (DLS) measurements (Malvern Zetasizer Nano ZS system).

## 1.4 Animal studies on antitumor activity of Iacs-eif3c-RNA

The human pharyngeal squamous carcinoma cell line FaDu cells were digested by trypsin and collected when reaching 90% confluence. And then FaDu cells were resuspended in serum-free medium mixed with 40% (v/v) Matrigel at a concentration of 2 × 10^7^ cells/ml. 2×10^6^ cells were inoculated into four-week-old female BALB/c nude mice through subcutaneous injection to construct tumor xenograft model. The shortest (W) and longest (L) diameter of the tumor were measured with calipers every two days. The tumor volume(mm^3^) was calculated with the formula: (W)^2^ × (L) × 0.5. On the 10^th^ day, mice were randomly assigned to treatment groups (n=5).

The tumor model mice were divided into three groups for Iacs-eif3c-RNA anti-tumor activity studies: treated with PBS group (PBS), treated with Au negative control group (Au-NC) and treated with Iacs-eif3c-RNA group (Iacs-eif3c-RNA). The tumor model mice were divided into five groups to evaluate the combination treatment of cisplatin and Iacs-eif3c-RNA: treated with PBS group (PBS), treated with Au negative control group (Au-NC), treated with Iacs-eif3c-RNA group (Iacs-eif3c-RNA), treated with PBS containing 0.25 mg/ml cis-platinum group (DDP) and treated with Iacs-eif3c-RNA containing 0.25 mg/ml cis-platinum group (Iacs-eif3c-RNA +DDP).

From day 10, mice were given 200 μL drugs by intraperitoneal injections every two days. Meanwhile, the body weight and tumor volume of mice were recorded. On the 20^th^ day after drugs treatment, all mice were sacrificed for the further experiments.

## 1.5 Inductively Coupled Plasma Mass

When the tumor volume reached ~200-300 mm^3^, 200 μL Iacs-eif3c-RNA was injected intraperitoneally to mice at 0, 4, 10 and 24 hours. Gold content in tumor tissues was determined using NexION 350D ICP-MS.

## 1.6 H&E staining, immunohistochemical (IHC) staining and TUNEL assay

Brain, heart, liver, lung, spleen, kidney and tumor were fixed in 4% paraformaldehyde for H&E staining. The fixed tumor tissues were used to determine the status of ki67, eIF3C and PD-L1 by IHC staining, and detect the cell apoptosis by TUNEL assay.

## 1.7 Toxicity studies

The C57BL/6 mice (6-8 weeks) were randomly divided into three groups for drug safety evaluation: treated with PBS group (PBS), treated with Au negative control group (Au-NC) and treated with Iacs-eif3c-RNA group (Iacs-eif3c-RNA). 200 μL PBS containing Iacs-eif3c-RNA was injected intraperitoneally to mice every two days, the mice were sacrificed for the following experiments on the 14^th^ day. The blood of mice was collected for blood routine test, including white blood cell (WBC), lymph (LYM), granulocyte (Gran), hemoglobin (HGB), red blood cell (RBC), and platelet (PLT). Besides, blood serum was collected to detect alanine aminotransferase (ALT), aspartate aminotransferase (AST), creatinine (CREA) and blood urea nitrogen (BUN) by using ELISA. Heart, spleen, liver, lung and kidney were removed for H&E staining.

## 1.8 IC50 of cis-platinum to FaDu cell line

FaDu cells were seeded into 96-well plates at 5000 cell/well. serial 2-fold dilutions of DDP were added after cell adhesion with final concentration at 0, 1.5625, 3.125, 6.25, 12.5, 25 and 50 μM. The cell viability was measured by Alamar Bue dye to convert into cell inhibitory rate of FaDu to cis-platinum.

## 1.9 Statistical analysis

ANOVA or two-way paired t test was used in all statistical analyses. *P* <0.05 was considered significant. Data were expressed as mean ± s.d.

# 2 Supplementary figures


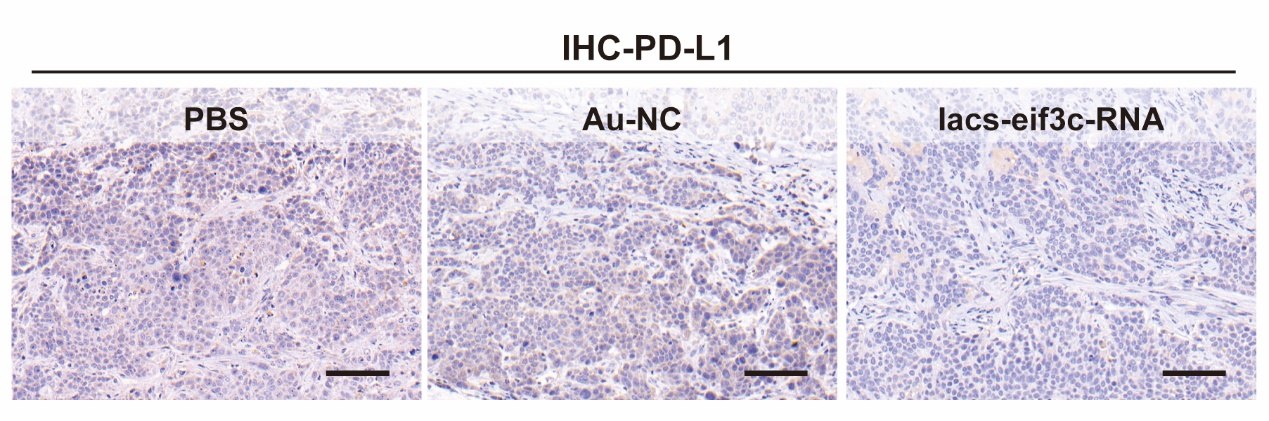


**Supplementary figure 1**. The representative histological IHC staining (PD-L1) of tumor in mice, treated with PBS, Au-NC and Iacs-eif3c-RNA. (magnification: 20×, scale bar: 100µm.)


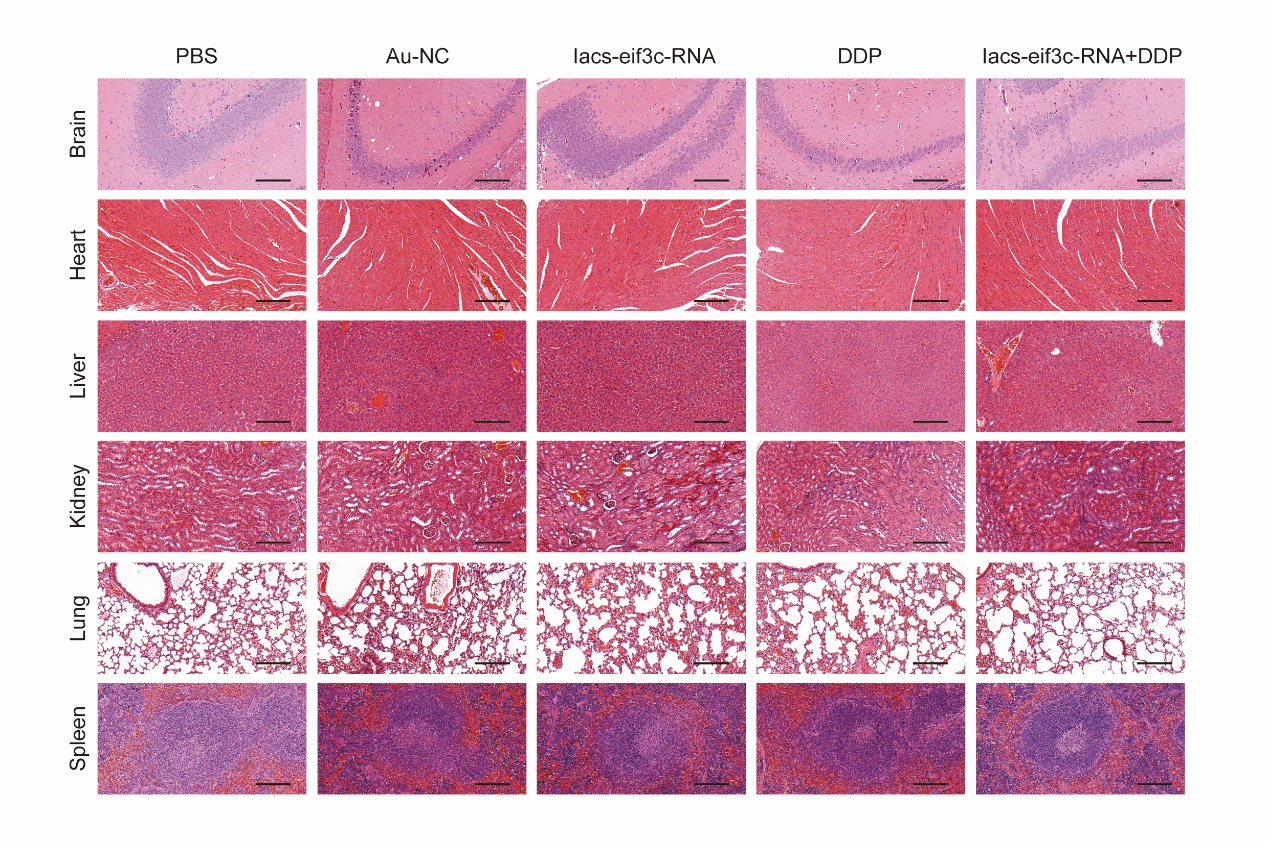


**Supplementary figure 2**. The representative histological H&E staining of brain, heart, liver, kidney, lung and spleen in mice, which were treated with PBS, Au-NC, Iacs-eif3c-RNA, DDP or DDP with Iacs-eif3c-RNA. (magnification: 20×, scale bar: 200µm.)
